# Case management for at-risk elderly patients in the English integrated care pilots: observational study of staff and patient experience and secondary care utilisation

**DOI:** 10.5334/ijic.850

**Published:** 2012-07-24

**Authors:** Martin Roland, Richard Lewis, Adam Steventon, Gary Abel, John Adams, Martin Bardsley, Laura Brereton, Xavier Chitnis, Annalijn Conklin, Laura Staetsky, Sarah Tunkel, Tom Ling

**Affiliations:** Cambridge Centre for Health Services Research, University of Cambridge, Forvie Site, Robinson Way, Cambridge CB2 0SR, UK; Partner, Ernst and Young LLP, 1 More London Place, London, SE1 2AF, UK; Nuffield Trust, 59 New Cavendish Street, London, W1G 7LP, UK; Cambridge Centre for Health Services Research, University of Cambridge, Forvie Site, Robinson Way, Cambridge CB2 0SR, UK; RAND Corporation, 1776 Main Street, Santa Monica, California 90401-3208, USA; Nuffield Trust, 59 New Cavendish Street, London, W1G 7LP, UK; RAND Europe Westbrook Centre, Milton Road, Cambridge, CB4 1YG, UK; Nuffield Trust, 59 New Cavendish Street, London, W1G 7LP, UK; RAND Europe Westbrook Centre, Milton Road, Cambridge, CB4 1YG, UK; RAND Europe Westbrook Centre, Milton Road, Cambridge, CB4 1YG, UK; Ernst and Young LLP, 1 More London Place, London, SE1 2AF, UK; Evaluation and Performance Measurement, RAND Europe Westbrook Centre, Milton Road, Cambridge, CB4 1YG, UK

**Keywords:** integrated care, older people, case management, patient experience, staff experience, hospital utilization, risk stratification, England

## Abstract

**Introduction:**

In 2009, the English Department of Health appointed 16 integrated care pilots which aimed to provide better integrated care. We report the quantitative results from a multi-method evaluation of six of the demonstration projects which used risk profiling tools to identify older people at risk of emergency hospital admission, combined with intensive case management for people identified as at risk. The interventions focused mainly on delivery system redesign and improved clinical information systems, two key elements of Wagner’s Chronic Care Model.

**Methods:**

Questionnaires to staff and patients. Difference-in-differences analysis of secondary care utilisation using data on 3646 patients and 17,311 matched controls, and changes in overall secondary care utilisation.

**Results:**

Most staff thought that care for their patients had improved. More patients reported having a care plan but they found it significantly harder to see a doctor or nurse of their choice and felt less involved in decisions about their care. Case management interventions were associated with a 9% increase in emergency admissions. We found some evidence of imbalance between cases and controls which could have biased this estimate, but simulations of the possible effect of unobserved confounders showed that it was very unlikely that the sites achieved their goal of reducing emergency admissions. However, we found significant reductions of 21% and 22% in elective admissions and outpatient attendance in the six months following an intervention, and overall inpatient and outpatient costs were significantly reduced by 9% during this period. Area level analyses of whole practice populations suggested that overall outpatient attendances were significantly reduced by 5% two years after the start of the case management schemes.

**Conclusion:**

Case management may result in improvements in some aspects of care and has the potential to reduce secondary care costs. However, to improve patient experience, case management approaches need to be introduced in a way which respects patients’ wishes, for example the ability to see a familiar doctor or nurse.

## Introduction

Healthcare systems are often ill-equipped to respond to the rapid rise in patients with multiple health problems [1–3]. Care for such people may become fragmented between different professionals and organisations, with attendant risks to quality and safety from duplication or omissions of care. This has led to widespread calls for care to be better integrated [4, 5]. Case management, a “proactive approach to care that includes case-finding, assessment, care planning and care co-ordination” [6] is a key feature of integration and is increasingly combined with use of tools to identify patients at risk of adverse outcomes [7].

In 2008, the English Department of Health invited applications from healthcare organisations offering innovative approaches to providing better integrated care following concerns that, especially for older people, care was becoming more fragmented. The proposals were intended “to achieve more personal, responsive care and better health outcomes for a local population” [8], but no blueprint was given on how integration was to be achieved. In 2009, sixteen integrated care pilots were appointed [9]. The sites took a wide range of approaches to integration which we have described in a separate report on all sixteen integrated care pilots [10]. The largest group of sites focusing on one type of intervention were the six which focused on intensive case management of elderly people at risk of emergency hospital admission. The reason for the focus on people at risk of emergency admission was because emergency admissions had been increasing and this was thought to represent a failure of care in the community as well as generating unnecessary secondary care costs. The six case management sites used a range of methods to identify older people at risk of admission including screening the elderly population with a risk profiling tool, e.g. PARR (Predicting And Reducing Readmission) or the ‘Combined Model’ [7]. People identified as at risk of admission were assigned a case manager, most often a nurse. In terms of the Chronic Care Model [3], a commonly used framework for planning quality improvement for chronic conditions, these sites focused mainly on delivery system design and improved clinical information systems, with a more limited focus on decision support and self-management support. The overarching theory behind these changes was broadly similar for all six sites, namely that better provision of primary care in the patient’s home could improve care for patients, avoid the need for specialist intervention and, in particular, avoid unscheduled or emergency admission to hospital [11, 12]. Here we report the outcome for the six case management sites, including staff reports of changes to their own work and to patient care, changes in patients’ experience, and changes in hospital utilisation and costs. A summary of the interventions is shown in the Box 1, with further details for each site in Table 1.

## Method

### Staff and patient experience

Questionnaires were sent to health and social care staff in the six sites in Summer 2010 (early in the evaluation period) and again in Spring 2011 (towards the end of the evaluation period). These included all staff closely involved in the pilot (e.g. case managers) and a random sample of staff from lists provided by the sites whose work might be impacted by the interventions (e.g. GPs, community nurses, social workers), with data analysed separately for these two groups. The survey asked about how staff thought their roles had changed from being in an ‘integrated care pilot’ and whether they thought care for their patients had improved as a result of pilot activities.

Patient questionnaires were sent out in autumn 2009 with a second questionnaire sent in autumn 2010. We only analysed responses from those patients who returned both questionnaires and who had a documented intervention which was delivered after the first questionnaire had been sent and at least two months before the second one. A sensitivity analysis was carried out on patients who reported no change in general health status between the two rounds to allow for the possibility that the natural history of decline in many of the conditions seen might influence patient responses.

We analysed both staff and patient questionnaires using conditional logistic regression allowing for clustering of respondents within sites to test for differences in responses in the two survey rounds. Further details of patient and staff selection are given in the appendix.

### Secondary care utilisation: individual patient level analysis

We analysed secondary care utilisation using data from Hospital Episode Statistics [13] (HES) which include up to fifteen diagnostic and procedural codes for all outpatient attendances and inpatient admissions in National Health Service (NHS) hospitals in England. Anonymised person level identifiers in HES data were generated by the NHS Information Centre for Health and Social Care using information supplied by the sites. Then, for patients who were documented to have received an intervention, we identified up to five controls from the national HES dataset matched for age, gender, ethnicity, area-level socio-economic deprivation using the Index of Multiple Deprivation [14], hospital utilisation in the previous year, diagnoses recorded in the previous three years, and predicted risk of future hospitalisation. Patients registered at primary care practices that were part of a pilot were excluded from being controls. Across all six sites, the analyses were based on 3646 patients confirmed to have received an intervention before September 2010 and 17,311 individually matched controls. The reasons for not being able to match controls for an additional 317 patients are documented in the appendix.

A difference-in-differences analysis was conducted to compare the two groups in terms of hospital utilisation in the six months before the intervention and the six months after. This analysis was carried out for emergency admissions, elective admissions, ambulatory care sensitive admissions, and attendance at outpatients. A concern in all matched control studies is that systematic differences might exist between intervention and control groups that are unobserved and therefore cannot be balanced between groups. We did find evidence of imbalance between the groups in this study despite the comprehensive nature of the matching, and in the appendix we describe simulation analyses which we conducted to estimate the likely effect of unobserved confounders (Appendix, section 3.1.1.). We also carried out a sensitivity analysis excluding site 2 which contributed more patients than any other site: the results of this sensitivity analysis were similar to the full analysis and are not reported here.

Notional secondary care costs were estimated by applying 2008/2009 Payment by Results tariffs to HES data. Activity not covered by the tariffs was costed using National Reference Costs. If neither tariff nor National Reference Costs were available, the activity was costed as the average tariff for the specialty under which the activity was coded. Although sites provided data on the additional costs of establishing the pilots, data were not available on the additional costs of providing new services, e.g. new case managers, so we focus in this paper on secondary care costs.

### Secondary care utilisation: practice level analysis

Practice level analyses were carried out for the same pre-specified groups of pilots as the individual patient analysis. We compared 117 practices (an average of 977,082 registered patients in any one year) which took part in an intervention with a random sample of half of all other practices in England. We used a longitudinal mixed effect Poisson regression model with a wide range of covariates to compare each of the two years before the pilot interventions with the two subsequent years. Whilst we could determine the exact date on which an intervention commenced for individual patients, for practices, we took the date the site entered the integrated care pilot scheme as the ‘start date’ and analysed data for the second full year of the scheme. This allowed the maximum time for interventions to have been introduced.

Staff and Patient questionnaire data were analysed using SPSS v19 and Stata v11. Patient and practice level analysis of secondary care utilisation was analysed using SAS v9.2. Further details of the methods are in the published protocol [15] and in the Appendix.

## Results

### Response rates

Questionnaires were sent to 276 members of staff in the first round, with response rates of 68.5% in the first round and 50.0% in the second. We did not have information to compare the characteristics of staff responding and not responding to the survey.

One thousand three hundred and eighty-five patient questionnaires were sent out in the first round with response rates in the two rounds of 65.8% and 47.7%. However, at the original time of sampling, sites could not be sure that all patients would receive an intervention and we therefore restricted our analyses to those 460 patients who returned both questionnaires and who subsequently were documented to have had an intervention delivered after the first questionnaire had been returned and at least two months before the second one was sent out. We were not able to compare the characteristics of patients responding and not responding to the survey; however, the mean age of respondents of 79.1 years (SD 8.1 years) was similar to the mean age of 79.6 (SD 11.5) for patients who were documented to have received an intervention across all sites and the gender breakdown was similar (54% female in respondents compared to 58.5% in all patients receiving an intervention).

Across all six sites, 3646 patients were confirmed to have received an intervention before September 2010 and we identified 17,311 matched controls for these.

### Staff questionnaires

Staff generally reported improved team working and communication, comparing responses early on and twelve months later in the evaluation period. For example in responses to the second questionnaire from 51 respondents whose work was directly involved with their pilot, 59.1% thought that they worked more closely with other team members, 67.5% that communication had improved within their organisation, and 65.0% that communication had improved with other organisations (compared to 4.5%, 7.5% and 2.5%, respectively who reported that these had got worse). Staff directly involved in their pilot also reported that the breadth and depth of their job had increased, that they had been given greater responsibility, and that they had more interesting jobs (46.3% more interesting, 51.2% no change, 2.4% less interesting).

In responses from 138 staff members from both groups to the question in the second survey “Have you seen improvements in patient care as a result of the Integrated Care Pilot?”, 56.7% of respondents replied ‘yes’, 14.9% ‘no’, 11.9% ‘not sure’, and 16.4% that it was ‘too early to tell’. However, comparing responses to the two survey rounds, fewer staff who had direct face-to-face contact with patients ‘strongly agreed’ with the statement “I am satisfied with the quality of care I give to patients” (41.9% before, 24.2% after, odds ratio 0.35, p<0.01). Full details of staff questionnaire responses, statistical tests and responses from different staff groups are available elsewhere [16].

### Patient questionnaires

Patients gave mixed responses about the care they had received. Following an intervention, they were no more likely to have had discussions with their doctor or nurse about how to deal with their health problems (87.3% in both rounds), but they were more likely to have been told that they had a care plan (30.5% vs. 22.8%, p<0.01). This increase in care plans occurred at a time when fewer patients were reporting in national surveys that they had received a care plan (data from the national GP Patient Survey shows an adjusted odds ratio or receiving a care plan of 0.95, CI 0.93–0.98 comparing surveys completed in autumn 2009 and autumn 2010). Patients in these case management sites were also more likely to report clear follow-up arrangements and to know whom to contact when discharged from hospital, and they were less likely to report having been given the wrong medicine in the preceding six months. However, they were also less likely to be able to see a doctor or nurse of their choice, felt less involved in decisions about their care, and were less likely to feel that their opinions and preferences had been taken into account (Table 2).

We considered the possibility that these responses reflected patients’ health having deteriorated over the 12 months between survey rounds. We therefore conducted additional analyses for 319 patients who reported no change in health status between the two rounds. This showed a similar picture with a preponderance of negative changes for care from GPs and social workers, but the changes in relation to nurses were no longer statistically significant. Responses including all non-significant findings and sensitivity analyses are shown elsewhere [16].

### Secondary care utilisation

Overall changes in secondary care utilisation at individual and practice levels are shown in Table 3. The individual patient level analysis shows a significant increase in emergency admissions and significant reductions in both elective admissions and outpatient attendances for intervention patients compared to controls. For the practice level analysis which includes all practice patients and not just the small proportion of registered patients who received an intervention, the only significant change is a reduction in outpatient attendance.

The apparent increase of 9% in emergency admissions in the individual patient analysis could have been due to imperfect matching between intervention patients and controls (e.g. intervention patients being sicker) and we have some evidence that this occurred because six month mortality was greater in intervention patients than controls (8.4% intervention patients, 4.8% controls in case management sites, Appendix Table A3). We therefore simulated the effect of an unobserved confounding variable and showed that a confounder would have to be almost twice as closely correlated with the outcome as the strongest known predictor of emergency admissions in order to reverse the apparent increase in emergency admissions (Appendix, section 3.1.1.). We conclude from this that while we cannot be certain by how much the pilot interventions increased emergency admissions, it is unlikely that they reduced them.

In the six months following an intervention, secondary care costs for individual patients were significantly increased for emergency admissions (increased by £172 per patient, CI £44–£300, p=0.01) but reduced for elective admissions (reduced by £329 per patient, CI £234–£424, p<0.001) and outpatient attendance (reduced by £66 per patient, CI £47–£84, p<001). Combined inpatient and outpatient costs were reduced by a mean of 9% in the six months following an intervention (£223 per patient, CI £54–£391, p=0.01).

## Discussion

When invited by the English Department of Health to produce innovations to integrate care more effectively, the government deliberately gave no guidance on how integration should be achieved, rather encouraging a range of diverse approaches to be developed ‘bottom up’ by those providing care. Although this produced a diverse range of interventions, a common approach adopted by pilot sites was case management of older people identified as being at risk of emergency hospital admission. In these interventions, the main integrating activities were between primary care practices and other community-based health services with a smaller number of pilots focusing on integrating primary care with secondary care or with social services. The evaluation represents one of a very small number of evaluations of case management combined with predictive risk modelling to identify patients at risk of hospital admission.

There are some clear results from the evaluation, though not all were ones that were originally intended. Staff were enthusiastic about their own involvement in the changes: their jobs became more interesting, they could observe better communication within and between organisations, and they could see patient care starting to improve. In addition, the sites documented a range of improvements in local evaluations which are not reported here, but are summarised elsewhere [10].

Patients were mixed in their views. More patients were told they had care plans and reported that their care was better organised following hospital admissions However, although patients have reported positive views about case management in previous studies [17–19], patients in this study were less likely to see a doctor or nurse of their choice and had less positive experiences of some key aspects of communication including being involved in decisions about their care. There may be a number of explanations for this, including the possibility of frail older people having to accustom themselves to new staff and the introduction of new routines of care. Most of the interventions involved appointment of new staff, so the elderly patients in this study may also have had to get used to new health professionals as well as new approaches to care. Continuity of care is important to patients, especially those with complex conditions [20], and patients included in this study appear to have experienced a reduction in continuity of care—i.e. they found it more difficult to see a familiar doctor or nurse. We speculate that the process of care planning and a more managed approach to care which was a key part of the interventions may have had the effect of ‘professionalising’ care rather than engaging patients more personally in their care. However, it is possible that patients would have settled down to the new approaches to care, and we might have found more positive results if we have been able to go back one or two years later.

Another unexpected finding was an increase in emergency admissions in the individual patient analysis relative to matched controls, especially as all six sites specifically aimed to reduce such admissions. This was despite individual staff reports of situations where emergency admissions had been avoided. Although some of the increase may have been an artefact of the matched control analysis used, we are confident from our sensitivity analyses that these sites did not achieve their aim of reducing emergency admissions beyond changes seen elsewhere. Previous studies have found some evidence that case management may reduce admissions among elderly people [21–23] although it may be more effective for a limited range of conditions such as heart failure [24, 25]. A previous evaluation of predictive risk modelling with case management in England was thought not to have reduced admissions in part because of using a case-finding model which did not identify people at sufficiently high risk of admission [26]; this led to the development of more sophisticated models such as those used in this study [7]. One possible explanation for the rise in emergency admissions may be that case management allowed pilots to be more alert to patients requiring hospital care. This would be an example of ‘supply induced demand’ where the provision of additional forms of care has the effect of identifying unmet health need—an effect which has been observed in other settings [27]. Our evaluation had no way of testing the appropriateness of the increased admissions that appear to have resulted from the case management interventions.

In contrast to the effect on emergency admissions, we found reductions in outpatient attendance and elective admissions, leading to a net reduction in combined inpatient and outpatient costs. The reduction in outpatient attendance could have been due to better coordination by community staff helping to reduce the need for unnecessary or duplicative outpatient appointments, or as part of planned changes to move services ‘closer to home’. However, the substantial reduction in elective admissions in case management sites was unexpected as this was not a key aim for any of the six sites. Over half the reduction appeared to be related to fewer admissions for people with cancer and half of these related to fewer admissions for chemotherapy even though similar proportions of patients had a recorded diagnosis of cancer in cases and controls (26.4% cases, 25.3% controls, Appendix Table A3). We are not able fully to explain this as cancer care was not a specific focus of the sites.

We found a net reduction in secondary care costs mainly because the reduction in elective admissions and outpatient attendances out-weighed the increased cost from emergency admissions. We were not provided with data to allow us to compare these costs with the additional costs of providing new services in primary care or in other sectors. Past literature does not show a consistent relationship between better coordination of care and cost reduction: a systematic review of interventions designed to improve care coordination found that they were most likely to improve health outcomes and user satisfaction (55% and 45% of studies, respectively) but costs were reduced in only 18% of studies reviewed [28, 29].

### Limitations of the study

There are challenges in drawing conclusions from this study. The pilots represent a somewhat heterogeneous group of interventions, and moreover they adapted and changed during the course of the pilot period, reflecting the changing health care environment in which they were operating. Our findings may therefore reflect a ‘real-life’ deployment of case management rather than the more artificial conditions of a randomised trial, but they do not allow us to describe a single simple intervention. In the absence of randomisation, the matched control method which we used to analyse secondary care by individual patients is subject, like all observational studies, to bias from unmeasured confounders. We found some differences in outcome between cases and controls evidenced by increase mortality in the intervention group. We assumed this was due to incomplete matching between cases and controls and therefore modelled the impact of unobserved confounders. Nevertheless, we cannot completely discount the possibility that the interventions increased mortality, and note that two recent high quality randomised controlled trials of interventions designed to prevent emergency hospital admission appeared to increase mortality in the intervention groups [30, 31]. The practice level analyses in our study are more robust to unmeasured confounding variables at individual patient level, but the weakness of this analysis is that any effect of the intervention will be diluted by the inclusion of large numbers of registered patients who were not subject to any intervention. For this reason, we suggest that the observed practice level reduction in outpatient attendance may be driven by broader changes in the sites rather than directly by the individual case management interventions. We used a range of sensitivity analyses, including simulation of the effect of confounding variables to draw what we believe are conservative conclusions. Nevertheless, we cannot completely avoid the bias that is inherent in this type of study design.

The results presented in this paper show that case management may result in improvements in some aspects of care and has the potential to reduce secondary care costs. However, case management approaches need to be introduced in a way which respects patients’ wishes, for example the ability to see a familiar doctor or nurse and has the potential to produce unexpected or negative consequences. The results are also disappointing in not meeting key objectives of the sites, despite the interventions generally including many of the elements of case management identified by Ross et al. [6].

We also note that a significant minority of staff thought that a two-year pilot period was not long enough to see whether care was improving. A recent report on an experiment to integrate care found that “two years of initial development followed by one year of live working” was required to show significant change [32]. Interventions designed to integrate care need to be monitored carefully and over the long-term to fully understand their impact on both patient experience and heath outcomes.

Overall, our evaluation shows that the link between using case management to improve care integration is not guaranteed to improve outcomes, and we have no reason to think this conclusion would be different for other countries or healthcare settings. This could be because the underlying programme theory is at fault, e.g. because supply induced demand increases appropriate admissions, or because the implementation of the interventions in this study was in some way faulty, a problem which Goodwin [33], Somme and Stampa [34] argue may be the cause of some case management interventions failing to produce their expected effects. It is also possible that our demonstration sites were implementing the wrong ‘type’ of integration—for example, well publicised approaches to integrated care in the US focus on vertical integration between primary and specialist care, and this was not a prominent feature of the English models. We were not able to distinguish between these explanations in this study, though in an accompanying paper [35] we do identify barriers and facilitators to the successful delivery of integrated care in these demonstration sites and provide a ‘routemap’ as a guide to the issues which need to be addressed when attempting to improve the integration of care.

## Statement of contribution made by individual authors

Martin Roland, Richard Lewis and Tom Ling were joint principal investigators on the project. They were responsible for the design and implementation of the evaluation. Adam Steventon, Gary Abel, Martin Bardsley and Xavier Chitnis were responsible for the design, data collection and matching of hospital utilisation data and for the analysis of hospital utilisation data. Annalijn Conklin and Laura Staetsky were responsible for the development, delivery and analysis of the patient and staff questionnaires and monitoring questionnaire returns from sites. John Adams was responsible for design and oversight of the statistical analysis. Laura Brereton was responsible for coordination of the study at RAND Europe including monitoring qualitative and quantitative data collection. Sarah Tunkel was responsible for coordination of the study at Ernst and Young including day-to-day liaison with sites and monitoring data collection. All authors have contributed to drafts of the paper. Martin Roland is guarantor of the paper.

## Funding and disclaimer

The study was funded by the Department of Health and was carried out by RAND Europe, the University of Cambridge and Ernst and Young. The views expressed are those of the authors and not of the Department of Health and does not constitute any form of assurance, legal opinion or advice. RAND Europe, the University of Cambridge and Ernst and Young shall have no liability to any third party in respect to the contents of this article.

## Ethical and governance approval

The study received ethics approval from Cambridgeshire 3 Research Ethics Committee, reference number 09/H0306/55. The National Information Governance Board confirmed that section 251 approval was not required to process patient data from Hospital Episode Statistics without consent, reference number ECC 4-15(a)/2009.

## Competing interests

The funding for this research was awarded by the Department of Health through a competitive peer reviewed process under a framework contract held by Ernst and Young. The research elements of the work described were carried out by RAND Europe, the University of Cambridge and the Nuffield Trust in close collaboration with Ernst and Young. The authors have no financial relationships with any organisations that might have an interest in the submitted work, and no other relationships or activities that could appear to have influenced the submitted work other than those declared below. Richard Lewis, as an Ernst and Young partner, has declared that Ernst and Young has been paid to deliver consultancy work unconnected to integrated care in two of the pilot sites (NHS Cumbria and Northumbria Healthcare NHS Foundation Trust). Ernst and Young is a consulting firm which may at times undertake consultancy work relevant to commissioning and provision of integrated care.

## Reviewers

**Nick Goodwin,** PhD, Senior Fellow, Health Policy, King's Fund, UK

**David Perkins,** PhD, Associate Professor, Director Centre for Remote Health Research Broken Hill Department of Rural Health University of Sydney, Australia

**Dominique Somme,** MD, PhD, Georges Pompidou European Hospital, University of Paris Descartes, Paris, France

## Figures and Tables

**Box 1:**
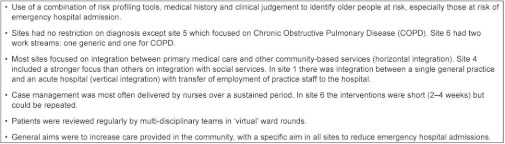
Key features of six English integrated care pilots focusing on case management of older people.

**Table 1:**
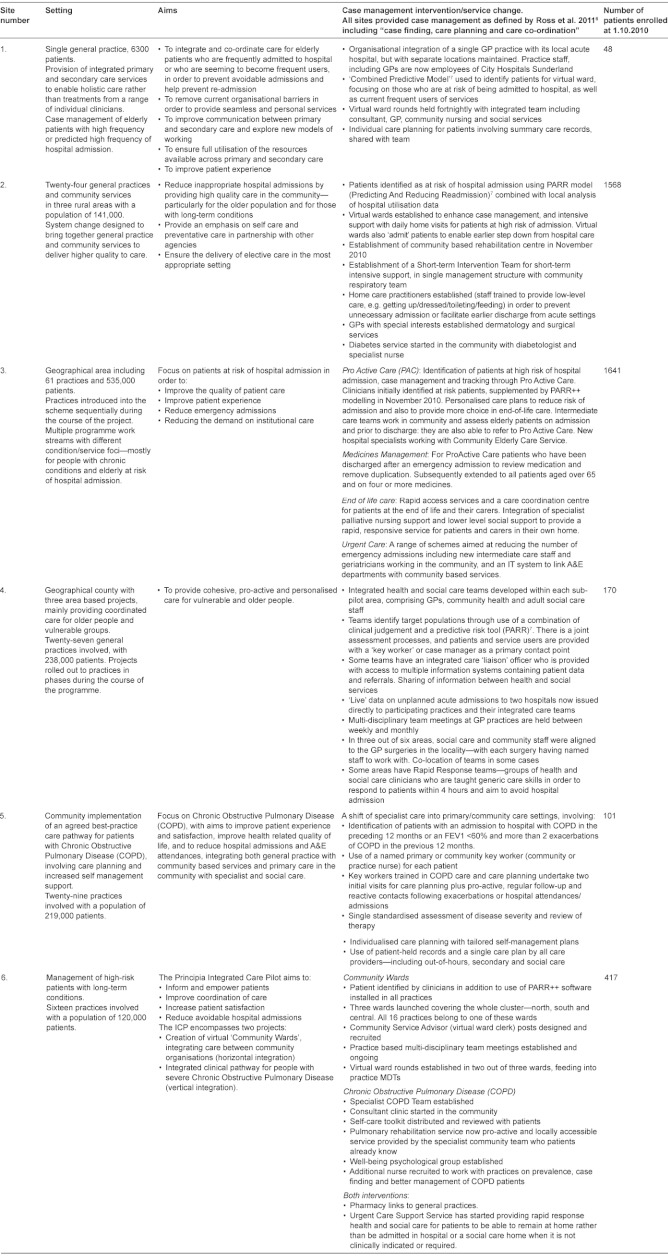
Details of six case management demonstration sites.

**Table 2:**
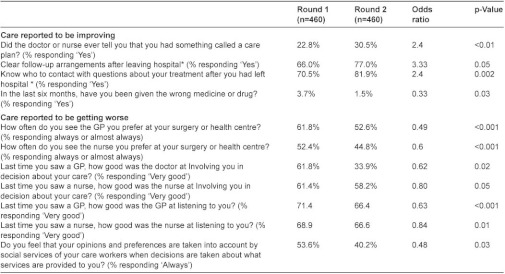
Summary of patient questionnaire responses (n=460).

*These two questions restricted to those reporting an admission in the previous six months.

**Table 3:**
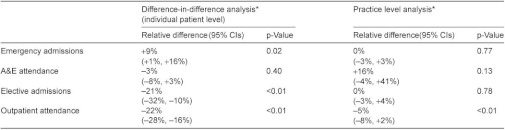
Changes in hospital utilisation for six case management.

*More detailed figures including absolute values are shown in Tables A5i and A5ii in the appendix.

**Table A1:**
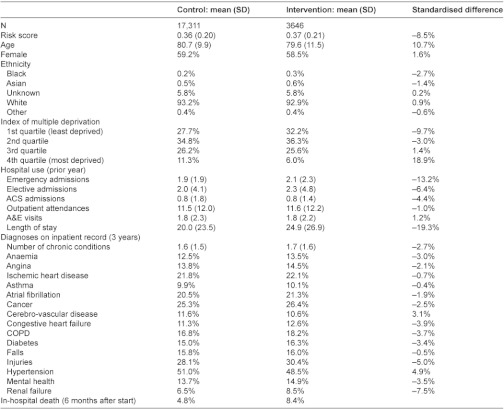
Characteristics of cases and controls in six case management sites

**Table A2:**
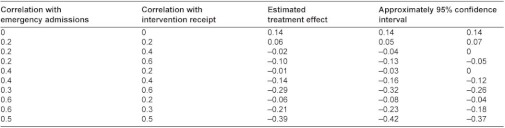
Correlation of the potential omitted confounder with intervention receipt and emergency admissions that would be required to eliminate the observed relationship.

**Table A3i:**
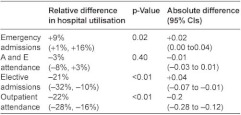
Individual patient analysis: changes in hospital utilisation comparing six months before with six months after an intervention.

**Table A3ii:**
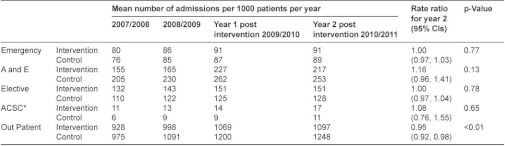
Practice based analysis: mean number of admissions per 1000 patients per year for intervention and control practices.

*ACSC=Ambulatory care sensitive condition.

## Case Management Pilots in England: observational study of staff and patient experience, secondary care utilisation and costs (Roland et al.) Appendix: Methods and additional statistical analyses

### Staff questionnaires

Survey data were collected from health and social care staff in all six pilots using a questionnaire administered in Summer 2010 (early in the intervention) and Spring 2011 (towards the end of the intervention). The questionnaire consisted of 24 questions on: personal experience of the piloted activity (e.g. changes to role, activities and work practices); views of health and social care quality received by patients/service users; communication within and between participating organisations as well as with other health and social care staff; experiences of team working, job satisfaction and ability to deliver high quality care; as well as information on individual background and demographic characteristics.

#### Sampling for staff questionnaire

The targeted sample was 50 members of staff per site, although some sites identified a smaller number (e.g. Church View which was a single practice site). Each site had a designated project manager who assisted in identifying the sample of staff participating in their pilot, providing a list of two groups:

- Members of staff formally associated with the pilot (in administrative or direct contact roles with service users), including all new appointees to the project and staff formally seconded full time or part time to the pilot; and
- Members of staff not formally associated with the pilot but whose work might be influenced in some way by pilot activity, such as GPs, community nurses, or social workers.

Group A was expected to include between 5 and 15 staff members per site, while group B in some cases exceeded the targeted number of 50. In such cases we randomly sampled the relevant number of staff from the second group so as to make a total of 50 for distribution.

We followed the same staff cohort for the repeated distribution of the questionnaire in spring 2011. Any new staff who had joined group A were included in the second round though in practice there were few of these. We also noted any staff that changed between groups A and B between survey rounds, although again such changes were rare. The numbers of questionnaires returned from the six sites were similar, and analyses conducted with and without allowing for clustering of responses within sites suggested that the findings (e.g. particularly positive or negative ones) were not dominated by the results from any one site. Two hundred and seventy-six questionnaires were sent out in round 1, with responses from 206 staff (73.9%) and 138 staff (50.0%) in the first and second rounds, respectively.

#### Analysis of staff questionnaire data

We used SPSS v19 to analyse the data from the ‘before’ time point (Summer 2010) and ‘after’ time point (Spring 2011). We dichotomised the response variables by coding the top response category (e.g. excellent or very good) or two top response categories as 1 and all other valid response items as 0.

Using STATA v11 we performed conditional logistic regression to test for changes in the responses of staff members responding in a particular way in the two survey rounds. As the number of staff responding from each site was small, we analysed the data aggregated from all sites. We adjusted the standard errors of regression coefficient for clustering of patients within sites, though this made no difference to the conclusions.

### Patient questionnaires

We created a survey instrument to assess the experience of patients/service users. Questionnaires were administered at two time points with one year in between: autumn 2009 and autumn 2010 (follow-up was repeated on the same sample of patients/service users). The questionnaire was developed using planned outcomes identified by pilot sites in their applications to join the scheme; a number of domains common to most pilots were included. The survey comprised 26 questions covering communication with primary care doctors and nurses; organisation and coordination of care; care planning; assessment of care from social services; arrangements following discharge from hospital; frequency of certain critical events (e.g. notes unavailable, test duplicated, wrong medication, wrong dose of medication, no follow-up arrangements after hospital discharge); and, type and frequency of recent health or social care provider.

Whenever possible we drew on existing validated instruments to select items to represent the identified domains including a number of questions from the English National GP Patient Survey (www.gp-patient.co.uk) Cognitive interviews with volunteer patients in Cambridge were used to test the questionnaire for construct validity before distribution.

#### Sampling for patient questionnaires

Sites identified a sample of up to 500 patients in each site. We planned to take a random sample in sites expecting more than 500, but the identified populations did not exceed this number in practice, and several small pilots identified 200 or fewer patients for inclusion. In these cases we sampled all patients who had received an intervention. For sites identifying patients/service based on their risk profile (rather than presence on a disease register), respondents were sampled as they were enrolled until the target of 500 was reached or until 31 March 2010 (a priori endpoint for enrolment). One thousand three hundred and eighty-five questionnaires were sent out in round 1, with responses from 912 patients (65.9%) and 661 patients (47.7%) in the first and second rounds, respectively. Six hundred and thirty-nine patients returned questionnaires in both rounds (46.1%).

When sites originally selected their sample of patients, they did not know whether they would all receive interventions within the evaluation period. In the end, only 460 of the 639 patients who returned both questionnaires had actually received a documented intervention two months or more before the second survey round and we therefore based our patient survey analysis on these 460 patients.

#### Analysis of patient questionnaire data

We used SPSS v19 to analyse the data from the ‘before’ time point (Autumn 2009) and ‘after’ time point (Autumn 2010). We dichotomised the response variables by coding the top response category (e.g. excellent or very good) as 1 and all other valid response items as 0. Using STATA v11 we performed conditional logistic regression to test for changes in responses between the two rounds of the survey. We also carried out separate analyses on subsets of patients whose self-reported health did not change between two rounds of survey and patients whose health changed (typically deteriorated) over the same period. We adjusted the standard errors for clustering of patients within sites, though this made little difference to the conclusions.

There were relatively more patients from one site (Cumbria) than from other sites in the case management group: we therefore conducted analyses for case management sites with and without patients from Cumbria. These analyses are not included in this report, but they did not alter the overall conclusions. As part of sensitivity analyses, we also coded the top two response categories (e.g. very good and good) as 1 and then the rest as 0, but found the results were not in general sensitive to the method of coding.

### Secondary care utilisation

#### Individual patient level analysis: selection and matching of controls

The following sections describe in more detail the approach used for data linkage, formation of control groups, and the difference-in-difference approach to the analysis of secondary care data.

Data linkage

Participants were linked at the person level to data on inpatient, outpatient, and accident and emergency activity sourced from the Hospital Episode Statistics (HES), a national data warehouse for England [1]. A HES and Office of National Statistics (ONS)-linked mortality file provided data on all deaths occurring in and out of hospital for those patients tracked through HES, although such data were only available for the pre-intervention period. The data linkage was conducted by the NHS Information Centre for Health and Social Care, which acted as a trusted third party and was the only organisation involved with the ICP evaluation to have access to both patient identifiers and data on secondary care activity. The National Information Governance Board confirmed that individual patient consent was not required for the data linkage to take place, and the approach was also scrutinised by the Cambridgeshire Ethics Committee.

The sites were asked to maintain a data management spreadsheet containing data for every person receiving one of their interventions, including the patient’s NHS number, date of birth, gender, post code, the date that the patient started to receive an integrated care pilot intervention, and the code of the GP practice with which they are registered. The spreadsheets were encrypted and transferred to the NHS Information Centre for data linkage. Two HES data linkage algorithms were then applied. The first pass of the algorithm required exact matches on NHS number and gender and a partial match on date of birth. Patients who were not linked following the first pass were then subject to a second pass that required exact matches on gender, date of birth and post code. After the data linkage had been conducted, the NHS Information Centre provided the Nuffield Trust with the HES IDs required to select the relevant records of hospital data from the HES data sets, together with information regarding the year of birth, gender, geographical area, intervention start date and practice code. No identifiable information or NHS numbers were transferred to the research team at any point in the data linkage process.

Sites maintained their data management spreadsheets throughout the pilot period, and the linkage was conducted three times at six monthly intervals on cumulative lists, including patient recruited to date. This enabled feedback to be provided to sites about the quality of the data recorded and maximised the proportions of participants that could be linked to HES.

Formation of matched control groups

Although there are several methods of selecting controls, the principle is always to select, from a wider population of potential controls, a subgroup of matched controls that is sufficiently similar to the intervention group with respect to baseline variables observed for all individuals. The selection of variables to incorporate in this process has been the subject of much debate. One case study and two sets of simulations show that including a variable that is related to recruitment into the intervention, but not to the outcome under study, does not improve bias in the estimated intervention effect, but can worsen the precision of the estimates [2, 3]. As a result we aimed to ensure that intervention and matched control patients were similar in terms of a set of variables that are known to predict future emergency hospital admissions [4]. This included age, gender, categories of prior hospital utilisation defined over a variety of time periods, number of outpatient specialties, the total number of chronic health conditions, area-level deprivation score (Index of Multiple Deprivation 2010 [5]), and 16 markers of specific health conditions (anaemia, angina and ischemic heart disease, asthma, atrial fibrillation and flutter, cancer, cerebro-vascular disease, congestive heart failure, COPD, diabetes, history of fractures, history of other falls, history of injury, hypertension, dementia, other mental health conditions, and renal failure). Health conditions were included regardless of whether they were recorded as the primary, secondary or other diagnosis, but we conducted further checks that intervention and matched control patients were similar in terms of recorded primary diagnoses.

Note that a fundamental limitation of the observational techniques being applied is that participants and matched controls may differ systematically according to some other, unobserved variable. This is known as ‘residual confounding’ and can only be avoided by a sufficiently large randomised trial. However, the variables used for the matching include some strong predictors of future hospital use.

Of the methods used to select matched controls, propensity score methods are perhaps the most established. These collapse baseline variables to a single scalar quantity known as the propensity score, which is the estimated probability of an individual receiving the intervention conditional on observed baseline variables [6]. A control is then selected on the basis that it has a similar propensity score to the individual receiving the intervention. More recently, prognostic score methods have been developed using a different scalar quantity, which is the estimated probability of an individual receiving the outcome (here, an emergency hospital admission) in the absence of the intervention conditional on observed baseline variables [7]. We chose the prognostic approach because the mechanism by which individuals had been selected for the interventions was known to have varied over time and between individual districts. A propensity score would have therefore been difficult to estimate in practice. In addition, the prognostic approach weights variables by how predictive they are of future hospital admissions. Since we were most concerned to balance variables that are strongly predictive of future hospital admissions, the prognostic approach helped us prioritise variables in the matching.

The formation of controls was limited to patients who had been linked to HES and began to receive an intervention before 30 September 2010. This cut-off point was chosen to ensure that at least six months of follow-up data were available within the timelines allowed for the evaluation. Importantly, controls were selected before follow-up data was available to the research team, to ensure no bias on behalf of the team.

In theory, controls could be chosen from within the integrated care pilot sites, from within similar areas, or nationally. Selecting controls from within the pilot areas ensures consistency of contextual factors relating to the configuration of services or characteristics of areas. However, it poses a number of risks, including the limited availability of controls and the possibility for the hospital utilisation of controls to be influenced by other aspects of the pilots. Such an approach may also increase the possibility for control and intervention patients to differ in terms of characteristics that are not recorded in operational data sets, if patients with these characteristics were strongly associated with recruitment into the interventions. Instead, we chose to select controls from outside of the pilot sites, and specifically from a pool of individuals registered in England but not registered at one of the general practices supplying patients for the pilot interventions. This resulted in a large number of individuals, and a random subset of 1–2 million individuals was selected, stratified by age and area-level deprivation score to match the characteristics of pilot participants. This was the pool from which matched controls was selected.

Patients were recruited into the interventions over a period of time stretching from February 2009 to the cut-off point of September 2010. We wanted to ensure our predictive risk scores reflected all hospital activity occurring before the interventions began, and further that they reflected the same period of time for controls as intervention patients. We therefore developed an algorithm that operated on a monthly basis and summarised individual histories over a range of periods. For example, when matching patients who began an intervention in February 2009, individual histories were created that summarised patterns of hospital use and recorded diagnoses up until 28 February 2009. A predictive risk score was calculated at 28 February for the subset of intervention patients that began an intervention in that month, as well as for the entire set of 1–2 million potential controls. This predictive risk score was then used in the subsequent steps of our matching algorithm. Note that the choice to summarise histories to the end of the month of intervention, rather than to the beginning of the month, meant that a limited amount of post-intervention data was included in the calculation of the risk scores. However, it meant that the predictive risk scores reflected all secondary care activity occurring before the start of the intervention. This was particularly important in some of the pilots where a substantial amount of activity was expected in the few days before intervention. In total the algorithm was run 18 times, as patients were not recruited in every month between February 2009 and September 2010.

Much of the data available on individual characteristics available for matching was sourced from hospital data. We therefore only aimed to construct matched controls for people with an inpatient or outpatient hospital contact within three years of the relative monthly end point. The same restriction was applied to the pool of potential controls.

The primary variable that we required to be similar between pairs of control and intervention patient was the predictive risk score. Several predictive risk models are in routine use in the NHS, but they do not relate to the specific population subgroup that is being considered here, namely patients with an inpatient or outpatient contact within a three-year period. For example, the Patients At Risk of Re-hospitalisation (PARR) model [4] produces predictions for patients with a recent inpatient admission, and the Combined Predictive Model produces predictions for entire registered populations [8]. We chose to create our own predictive model using a similar set of predictor variables to PARR, but calibrated to the patterns of care observed in the integrated care pilot sites for patients with an inpatient or outpatient contact within a three-year period. The models were rebuilt for every month of the algorithm using pooled data from all of the sites, so that 18 models were built in total. Intervention participants were excluded when fitting the predictive risk models in line with recent recommendations for prognostic matching [7]. A split-sample model development approach was adopted, so that the data set was split at random, with one half used to develop models that could be tested against the other half of the data set. A&E data were not available to use as predictor variables for the model. Having fit the models, risk scores were calculated for the intervention patients and potential controls. Matching was performed for one intervention patient at a time. The precise method was iterated until satisfactory balance was achieved between intervention and matched control patients on the set of variables described above. We measured balance by the standardised difference. This is defined as the difference in the sample means as a percentage of the square root of the average of the sample variances. While there is no clear consensus on the issue, some researchers have proposed that a standardised difference of >10% denotes meaningful imbalance in the variable [9]. As the standardised difference only measures a difference in means, the other metrics including Q-Q plots were used to compare the distribution of covariates.

In the final version of the algorithm, the pool of potential controls was successively limited in a series of steps. To begin with, it was reduced to those of the same combination of discrete variables (for example, gender) as the intervention patient and with a similar predictive risk score, defined as a logit within 20% of a standard deviation [6], with the predictive risk score calculated at the end of the month of intervention. Histories of hospital use and diagnoses of major disease groups were then recalculated for the intervention patient and the remaining set of potential controls using the precise date that the patient in question received the intervention. At this stage, individuals who had died before the intervention start date were also excluded from being a control. Matching was then performed simultaneously according to a key set of variables including the predictive risk score, age, area-level socio-economic deprivation (based on the Index of Multiple Deprivation 2010), number of chronic health conditions, prior number of emergency admissions, elective admissions, outpatient attendances, and days in hospital. The five closest controls according to the Mahalanobis distance were retained [10]. Controls were selected without replacement so that the same individual could not act as a control to more than one intervention patient. Balance was assessed using the entire set of variables selected at the outset of the project.

The control matching was performed by the Nuffield Trust, and the final set of matched controls was discussed and agreed by the wider research team prior to the availability of follow-up data. Across all six sites, we were able to find controls for 3646 of 3963 patients confirmed to have received an intervention before September 2010, and for these we identified 17,311 individually matched controls. Reasons that controls could not be found were:

1. Index patient not linked to HES
2. No prior hospital use (therefore no data to use in the matching)
3. Well-matched controls could not be found

The characteristics of intervention patients and controls in the six case management sites is shown in Table A1.

Comparison of endpoints

Analysis of inpatient activity was restricted to ordinary admissions, excluding transfers and regular attendances and maternity events (patient classifications 1 and 2 only). Admissions were classified further based on defined admission methods into emergency activity (codes 21–28) and elective activity (all other codes excluding transfers). Bed days included stays following emergency and elective admissions, with same day admissions and discharges assigned a length of 1 bed day. Outpatient activity was restricted to appointments that were attended (codes 5 and 6). Our set of ambulatory sensitive conditions was derived from AHRQ and Purdy et al. [11, 12] and described in the published study protocol Ling et al. [13]. Analysis of accident and emergency activity included all visits, regardless of subsequent inpatient admission, but was limited to April 2007 to March 2010 due to the available data. Since the HES-ONS linked mortality file was only available for the pre-intervention period, comparisons of mortality post intervention were restricted to analysis of deaths occurring within hospital only.

Notional costs of care were estimated from HES data by applying the set of mandatory and indicative tariffs used in England for the reimbursement of inpatient and outpatient care (2008/2009 Payment by Results tariffs). These assume a stay of a certain number of days (the ‘trim point’), and allow hospitals to charge a pre-specified amount for each additional excess bed day. Costs were not adjusted for the regional costs of providing care, and so were effectively a weighted activity measure which allowed robust comparison of the magnitude of care received for control and participants. Activity not covered by the tariffs was costed using the National Reference Costs (NRC). If neither tariff nor NRC were available, the activity was costed as the average tariff for the specialty under which it was delivered.

A ‘difference-in-difference’ analysis was conducted for each endpoint, which compared the two groups in terms of the differences between the numbers of admissions in the six months after the date of the intervention to the numbers in the six months before. This aimed to reduce the impact of any unobserved systematic differences between the groups.

The difference-in-difference analysis was conducted using a linear model with the metric as the dependent variable and three predictors related to whether the observation was before or after intervention, whether it was for the control or intervention group, and the interaction of the two. Hospital use for individuals in the same site will tend to be correlated. This within-site homogeneity was accounted for in the analysis by constructing hierarchical difference-in-difference models which included random effects at the site level. The matched nature of the data was also taken account using a random effect for each ‘block’, consisting of an intervention patient and their matched controls.

##### Estimating the effect of an unobserved confounding variable

The increase in emergency admissions observed in pilot sites (and case management sites in particular) could have been due to imperfect matching between cases and controls, e.g. cases being sicker and hence more likely to be admitted. Although cases and controls were similar in terms of the variables that we could observe, it is nevertheless possible that systematic unobserved differences existed between the groups. We have some evidence that this was the case because six-month mortality was greater in cases than controls (8.4% vs. 4.8% in case management sites, see Table A1), an effect that was unlikely to be caused by the interventions. In order to estimate the effect of incomplete matching, we performed an additional analysis by using a simulation technique outlined by Higashi et al. [14]. This involved making assumptions about the strength of an omitted confounder variable, and then estimating what impact controlling for that variable would have had on the analysis of emergency admissions.

We simulated a continuous confounder based on a range of assumptions about the correlation with emergency admissions and recruitment into the intervention. In each scenario, the variable was simulated using a rejection sampling approach, generating triads of (U, T, Y) that met the following criteria:

1. Allocation into intervention, T ∼ Bernoulli (0.5)
2. Emergency admissions in the six months post intervention, Y ∼ according to the observed marginal distribution.
3. Unobserved confounder, U was a mixture of normal distributions.
4. Correlation (U, T) and correlation (U, Y) as desired

We continued to generate these triads until T=t, Y=y, the values observed in the data. We then used OLS regression to estimate the effect of the intervention adjusting for the simulated values of the unobserved confounder.

From Table A2, we selected correlation values to illustrate the magnitude of the correlations required to turn no effect into an increase of 9% in emergency admissions (as found in case management sites). It can be seen that, for a reduction in emergency admissions to have been masked, an unobserved confounder would have had to exist with high correlations, for example of 0.4 with both the intervention and with emergency admissions.

Finally, we considered how likely it was that such a confounder would exist. We know from our data that the strongest predictor of future emergency admission is a past history of emergency admission, which has a correlation of 0.25 with emergency admissions and 0.10 with intervention receipt. Therefore for a hypothetical confounder to turn even a very small reduction in admissions into a 9% increase would require a confounder correlated almost twice as strongly with the outcome as the strongest predictor we know to date. We consider this unlikely and therefore conclude that it is unlikely that a confounding variable masked a true reduction in emergency admissions over six months among patients in case management sites. While we cannot be certain the extent to which pilot interventions were associated with increased admissions in the intervention group, it is unlikely that the interventions reduced emergency admissions.

#### Practice level analysis

While the individual patient-based analysis will give the most direct measure of the effectiveness of the interventions, it is still of interest to see if the effect of the intervention can be seen at the practice level. While practice based analyses are more robust to unmeasured covariates at individual patient level, any effect of the intervention is greatly diluted by individuals who are not exposed to the intervention.

In this analysis, we have separately used the number of elective admissions, the number of emergency admissions, the number of ambulatory care sensitive conditions, the number of outpatient attendances, and the number of A&E attendances recorded in HES aggregated at practice level. For each practice the data were aggregated into 14 age by gender groups (age groups 0–4, 5–14, 15–44, 45–64, 65–74, 75–84 and 85+). Practices containing patients who received an intervention as part of the ICP scheme were compared to a random selection of half of all other practices in England. This comparison was made for the two years following intervention (12 months from 1.4.09 and 12 months from 1.4.10), expecting a greater effect in the second year. Note that due to the unavailability of data, the A&E attendances analysed are only for 11 months in the final year.

The analysis performed was a longitudinal mixed effect Poisson regression using four years of data (two years prior to and two years following the intervention) employing a difference in differences methodology. The regression analysis controlled for the following covariates: list size for each year under study; patient age and gender profile; list size per FTE GP; mean IMD; patient ethnicity profile; QOF quality scores; QOF prevalence scores; mean years since qualification of GPs; the proportion of GPs who qualified in the UK; and the Low Income Scheme Index (LISI) score [15]. The random effects are included so that the underlying admission rate in each practice is accounted for and that this rate can change year on year. This is achieved by fitting an unstructured covariance matrix. An interaction term between year (following intervention) and intervention group allows us to assess the effect of the intervention in the two years following intervention. Practices with <1000 patients in any year were excluded from the analysis as were all data from individual practices with list size changes of more than 10% in any one year.

#### Analysis of secondary care costs

For secondary care utilisation comparing patients/service users with controls, notional costs of care were estimated from HES data by applying the set of mandatory and indicative tariffs used in England for the reimbursement of inpatient and outpatient care (2008/2009 Payment by Results tariffs). These assume a stay of a certain number of days (the ‘trim point’), and allow hospitals to charge a pre-specified amount for each additional excess bed day. Costs were not adjusted for the regional costs of providing care, and so were effectively a weighted activity measure which allowed robust comparison of the magnitude of care received for control and participants. Activity not covered by the tariffs was costed using the National Reference Costs (NRC). If neither tariff nor NRC were available, the activity was costed as the average tariff for the specialty under which it was delivered.

#### Supplementary tables

Tables A3i and A3ii are expansions of Table 2 in the main paper to include absolute values and confidence intervals. Table A3i shows the values for the individual patient analysis and Table A3ii for the practice based analysis for those practices included in the individual patient analysis.

## References

[1] Salisbury C, Johnson L, Purdy S, Valderas JM, Montgomery AA (2011). Epidemiology and impact of multimorbidity in primary care: a retrospective cohort study. British Journal of General Practice.

[2] Glynn LG, Valderas JM, Healy P, Burke E, Newell J, Gillespie P (2011). The prevalence of multimorbidity in primary care and its effect on health care utilization and cost. Family Practice.

[3] Coleman K, Austin BT, Brach C, Wagner EH (2009). Evidence on the Chronic Care Model in the new millennium. Health Affairs (Millwood).

[4] Curry N, Ham C (2010). Clinical and service integration. The route to improved outcomes.

[5] Shaw S, Rosen R, Rumbold B (2011). What is integrated care. An overview of integrated care in the NHS.

[6] Ross S, Curry N, Goodwin N (2011). Case management. What is it and how can it best be implemented?.

[7] Lewis G, Curry N, Bardsley M (2011). Choosing a predictive risk model: a guide for commissioners in England.

[8] Department of Health (2008). Next stage review. Our vision for primary care.

[9] Department of Health (2009). Integrated care pilots: an introductory guide.

[10] RAND Europe, Ernst and Young (2011). National evaluation of the DH integrated care pilots. Final report.

[11] Billings J, Zeitel L, Lukomnik J (1993). Impact of socioeconomic status on hospital use in New York City. Health Affairs.

[12] Association for Healthcare Quality and Research (2004). Guide to prevention quality indicators: hospital admission for ambulatory care sensitive conditions. Revision 3.

[13] Hospital Episode Statistics (2005–2012). http://www.hesonline.nhs.uk/.

[14] Department of Communities and Local Government (2010). Index of multiple deprivation 2010.

[15] Ling T, Bardsley M, Adams J, Lewis R, Roland M (2010). Evaluation of UK integrated care pilots: research protocol. International Journal of Integrated Care [serial online].

[16] RAND Europe (2012). National evaluation of DH integrated care pilots. Appendix H: detailed results of patient and staff surveys.

[17] Sheaff R, Boaden R, Sargent P, Pickard S, Gravelle H, Parker S (2009). Impacts of case management for frail elderly people: a qualitative study. Journal of Health Services Research and Policy.

[18] Elwyn G, Williams M, Roberts C, Newcombe R, Vincent J (2008). Case management by nurses in primary care: analysis of 73 ‘success’ stories. Quality in Primary Care.

[19] Brown K, Stainer K, Stewart J, Clacy R, Parker S (2008). Older people with complex long-term health conditions. Their views on the community matron service: a qualitative study. Quality in Primary Care.

[20] Aboulghate A, Abel A, Elliott M, Parker R, Campbell J, Lyratzopoulos G Do English patients want continuity of care, and do they receive it?. British Journal of General Practice.

[21] Hutt R, Rosen R, McCauley J (2004). Case-managing long-term conditions. What impact does it have in the treatment of older people.

[22] Low L-F, Yap M, Brodaty H (2011). A systematic review of different models of home and community care services for older persons. BMC Health Services Research.

[23] Baker A, Leak P, Ritchie L, Lee A, Fielding S (2012). Anticipatory care planning and integration: a primary care pilot study aimed at reducing unplanned hospitalisation. British Journal of General Practice.

[24] McAlister F, Stewart S, Ferrua S, McMurray J (2004). Multidisciplinary strategies for the management of heart failure patients at high risk for admission a systematic review of randomized trials. Journal of the American College of Cardiology.

[25] Gonseth J, Guallar-Castillón P, Banegas J, Rodríguez-Artalejo F (2004). The effectiveness of disease management programmes in reducing hospital re-admission in older patients with heart failure: a systematic review and meta-analysis of published reports. European Heart Journal.

[26] Gravelle H, Dusheiko M, Sheaff R, Sargent P, Boaden R, Pickard S (2007). Impact of case management (Evercare) on frail elderly patients: controlled before and after analysis of quantitative outcome data. British Medical Journal.

[27] Department of Health and Ageing (2007). The National Evaluation of the second round of Coordinated Care Trials Australian Government. http://www.health.gov.au/internet/main/publishing.nsf/Content/pcd-chronic-coordinated-care-round-2-trials.

[28] Powell Davies G, Harris M, Perkins D, Roland M, Williams A, Larsen K (2006). Coordination of care within primary health care and with other sectors: a systematic review.

[29] Powell Davies PG, Williams AW, Larsen K, Perkins D, Harris MF, Roland M (2008). Coordinating primary health care: an analysis of the outcomes of a systematic review. Medical Journal of Australia Medical Journal of Australia.

[30] Takahashi P, Pecina J, Upatising B, Chaudhry R, Shah N, Van Houten H (2012). A randomized controlled trial of telemonitoring in older adults with multiple health issues to prevent hospitalizations and emergency department visits. Archives of Internal Medicine.

[31] Fan VS, Gaziano JM, Lew R, Bourbeau J, Adams SG, Leatherman S (2012). A comprehensive care management program to prevent chronic obstructive pulmonary disease hospitalizations: a randomized, controlled trial. Annals Internal Medicine.

[32] Shaw S, Levenson R (2011). Towards integrated care in Trafford.

[33] Goodwin N (2011). Reviewing the evidence on case management: lessons for successful implementation. International Journal of Integrated Care [serial online].

[34] Somme D, Stampa M (2011). Ten years of integrated care for the older in France. International Journal of Integrated Care [serial online].

[35] Ling T, Brereton L, Conklin A, Newbould J, Roland M (2012). Barriers and facilitators to integrating care: experiences from the English integrated care pilots. International Journal of Integrated Care [serial online].

